# Liver transplantation in adult polycystic liver disease: the Ontario experience

**DOI:** 10.1186/s12876-021-01703-x

**Published:** 2021-03-09

**Authors:** Mohammed Alsager, Shuet Fong Neong, Radhika Gandhi, Anouar Teriaky, Ephraim Tang, Anton Skaro, Karim Qumosani, Les Lilly, Zita Galvin, Nazia Selzner, Mamatha Pallavi Bhat, Klajdi Puka, Mayur Brahmania

**Affiliations:** 1 Division of Gastroenterology and Multi-Organ Transplant, Western University, London, ON Canada; 2 Multi-Organ Transplant Program, University Health Network, Toronto, ON Canada; 3 Department of Epidemiology and Biostatistics, Western University, London, ON Canada; 4 Room A10-224; London Health Sciences Centre: University Hospital, Western University, London, ON N6A 5A5 Canada

**Keywords:** Polycystic liver disease, Liver transplantation, PCLD outcome

## Abstract

**Background:**

Liver transplantation (LT) remains the curative treatment for symptomatic Polycystic Liver Disease (PCLD) patients and is associated with excellent survival rates. The aim of the study is to review the Ontario experience in LT for PCLD.

**Methods:**

A retrospective study was performed from pre-existing LT databases from the LT Units at Toronto General Hospital and London Health Sciences Center, which are the two LT programs in Ontario, Canada. This database contains demographic, clinical parameters and follow-up of all patients transplanted for PCLD. Data was extracted for patients who underwent LT between January 2000–April 2017 and included follow up until December 31st, 2018.

**Results:**

A total of 3560 patients underwent LT, of whom 51 (1.4%) had PCLD and met inclusion criteria. 43 (84%) of these patients were female. The median physiologic Model for End Stage Liver Disease (MELD-Na) score at time of referral was 13 (IQR = 7–22), however all patients required MELD-Na exception points to receive LT. The median age of transplant was 62 years (IQR = 59–64) for male vs. 52 (IQR = 45–56) for female patients. 33 (65%) of our cohort had PCLD while 9 (17.5%) had ADPKD and 9 (17.5%) had both diseases. 39 (76%) had LT due to symptoms of mass effect, while 8 (16%) had portal hypertensive complications. After a median follow-up of 6.3 (IQR = 2.9–12.5) years, the probability of survival was 96% (95% CI: 90%, 100%). Log-rank test, comparing survival analysis between males and females did not show a statistically significant difference (p = 0.26).

**Conclusion:**

Most patients underwent LT for PCLD due to symptoms of mass effect with women being more likely than men to undergo LT. LT for PCLD had excellent long-term survival.

## Background

Polycystic liver disease (PCLD) can occur in isolation or, more commonly, as an extra-renal manifestation of autosomal dominant adult polycystic kidney disease (ADPKD). For the group of patients that develop only renal cysts at diagnosis, 30% can develop liver cysts within 30 years of diagnosis and the prevalence is estimated to be 1:5000 [1–3]. In patients with ADPKD, PCLD is diagnosed when there are more than 20 cysts present and more than 94% of those aged 35 years and above will have hepatic cysts as the size increases with age (1–3%/year) [4]. Other risk factors associated with severe cystic disease in ADPKD include female gender, exogenous female hormones, multiple pregnancies and a greater burden of renal cysts.

As hepatic cysts enlarge, they cause a mass effect and exert pressure on adjacent organs. As a result, patients experience dyspnea, early satiety, weight loss and malnutrition [4]. Portal hypertension can also arise as large hepatic cysts cause architectural distortion, increasing intrahepatic resistance to blood flow. Hepatic venous outflow obstruction and direct portal vein compression also contribute to the mechanism of portal hypertension in this patient group [4]. Liver transplantation (LT) is the definitive curative treatment for PCLD patients with excellent patient survival rates when compared to patients transplanted for other indications. The indication for Liver transplant (LT) is usually for symptomatic relief. Once listed, Patients with PCLD are awarded exceptions MELD-Na points every three months. In this study, we examined the Ontario experience with regards to LT in PCLD patients.

## Methods

### Study design, inclusion and exclusion criteria

**Fig. 1:**
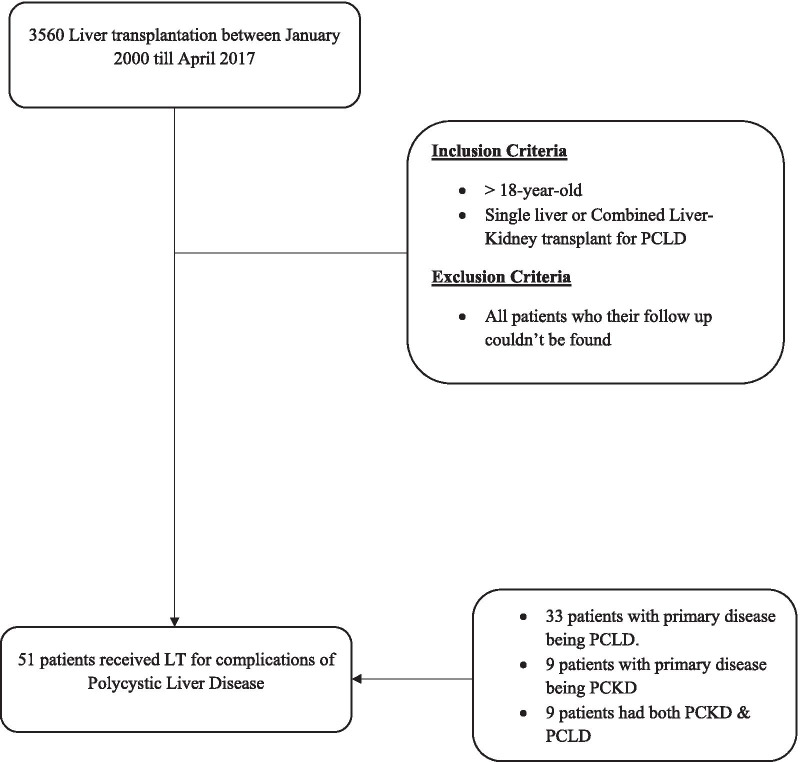
Patient selection

A multi-center retrospective cohort study was performed of patients diagnosed with PCLD and referred for LT between January 1, 2000 and April 30, 2017. The study was conducted at the two LT centers in the province of Ontario (University Health Network, Toronto; and University Hospital; London) which serve an estimated population of 14,000,000 and perform approximately 225 LT per year (including living donor and simultaneous liver kidney (SLK)). The diagnosis of PCLD was based on radiographic evidence (CT or MRI). Patients who were transplanted (single organ, SLK, or living donor) for complications related to PCLD were included in the study. Patients were excluded from LT if there had any contraindications such as poor social support, frailty or uncontrollable psychiatric illness. All data was extracted for patients who had follow up till December 31st, 2018. We also excluded patients whom follow-up data is unavailable in the electronic health records. Figure 1 shows the final patients selection. The study was conducted in accordance with the guidelines of the Declaration of Helsinki and the principles of Good Clinical Practice and is approved by the ethical review boards of University of Toronto and Western University.

### Statistical analysis

Continuous variables were expressed as median values with interquartile ranges (IQR) and categorical variables were expressed as frequencies (percentages). Continuous variables were compared between groups using the Wilcoxon rank-sum test and categorical variables were compared using Fisher’s exact test. The starting time point, or baseline, for the analysis was defined as the date a patient received LT. Patients were followed until death or date of the last outpatient visit. Data was analyzed using the R statistical software (Version 3.2.2).

## Results

### Patient characteristics

There was a total of 3560 LT performed during the study period with 51 (1.4%) related to PCLD and meeting inclusion criteria. 43 (84.3%) LT recipients were female, while 8 (15.6%) recipients were male. The median age for all patients was 54 years (IQR = 46–59). In subgroup analysis, the median age for female recipients was 52 years (IQR = 45–56) vs. a median age of 62 years in male recipients (IQR = 59–64). The median waitlist time was 9.7 months (IQR = 5.5–18.1). The median physiologic MELD score at the time of transplant was 13 (IQR = 7–22). The median physiologic MELD score for male population was 22 (IQR = 17–22) in comparison to a median physiologic MELD of 10 (IQR = 6–21). In the male population, 7 (87%) had worsening kidney function with a median creatinine of 426 (IQR = 237–485) as oppose to female group 15 (34.8%) which had a median creatinine of 91 with (IQR = 73–184). However, all patients were transplanted with exception points. 39 (76%) were referred for LT, as they were experiencing mass effect symptoms such as early satiety, abdominal pain, nausea and vomiting and 8 (16%) had portal hypertension related complications as the main indication for LT. The remaining 4 (8%) patients’ indications of transplant were not documented. In patients with portal hypertension, 5 (62.5%) had portal hypertension due to vascular invasion while the remaining 3 (37.5%) had portal hypertension due to fibrotic parenchyma. All patients had registered dietitian involved in their as part of transplant team however, none of the patients required additional nutritional support i.e., TPN. Table 1 show the demographic characteristics of our cohort.

**Table 1:** Patient demographics of those undergoing liver transplantation for polycystic liver disease

Variable	Total	Male	Female
Number of Transplants	51 (100%)	8 (16%)	43 (84%)
Median age*	54 (46–59)	62 (59–64)	52 (45–56)
Median Waitlist (months)	9.8 (5.5–18.7)	10.2 (7.4–14.1)	9.5 (5–18.8)
Median MELD score at time of transplant	13 (7–22)	22 (17–22)	10 (6–21)
Median (years) follow up	6.3 (12.5)	2.9 (2.2–4.1)	8 (3–13.8)
Orthotopic Liver transplant	46 (90.1%)	8 (15.6%)	38 (74.5%)
Living donor	5 (9.8%)	0 (0%)	5 (9.8%)
Liver transplant alone	29 (56.8%)	1 (1.9%)	28 (54.9%)
Kidney first then liver transplant	15 (29.4%)	3 (5.8%)	12 (23.5%)
Simultaneous Liver-Kidney transplant (SLK)	7 (13.7%)	4 (7.8%)	3 (5.8%)
Median Creatinine $(\upmu$ mol)	101 (80–319)	426 (237–485)	91 (73–184)
Median Hemoglobin (g/L)	116(109–126)	118 (113–127)	116 (109–127)
Median platelet (10^9^/L)*	189 (130–239)	189 (118– 203)	193 (132–240)
Median Bilirubin $\upmu$ mol*	10 (8–14)	10 (9–11)	10 (7–14)
Median INR*	1.1 (1–1.2)	1.2 (1.15–1.2)	1.1 (1–1.2)
Median Albumin (g/L)*	40 (35–43)	36 (33–40)	40 (35–43)
Death	4 (7.8%)	1 (1.9%)	3 (5.8%)

^*^Medians expressed as IQR

### Main disease and type of LT

33 (64.7%) patients had PCLD as the primary disease, 9 (17.6%) had polycystic kidney disease (PCKD) as their primary disease and 9 (17.6%) patients had both polycystic liver and kidney disease. 29 (56.9%) received LT alone, 7 (13.7%) had a simultaneous liver kidney transplant (SLK) and 15 (29.4%) patients received a kidney transplant followed by LT. There were 46 (90%) orthotopic LT and 5 (10%) living donor LT. Out of patients who received orthotropic LT, 38 (82.6%) were female. All patients who received living donor LT were females.

### Post-operative management, complications and survival

**Fig. 2:**
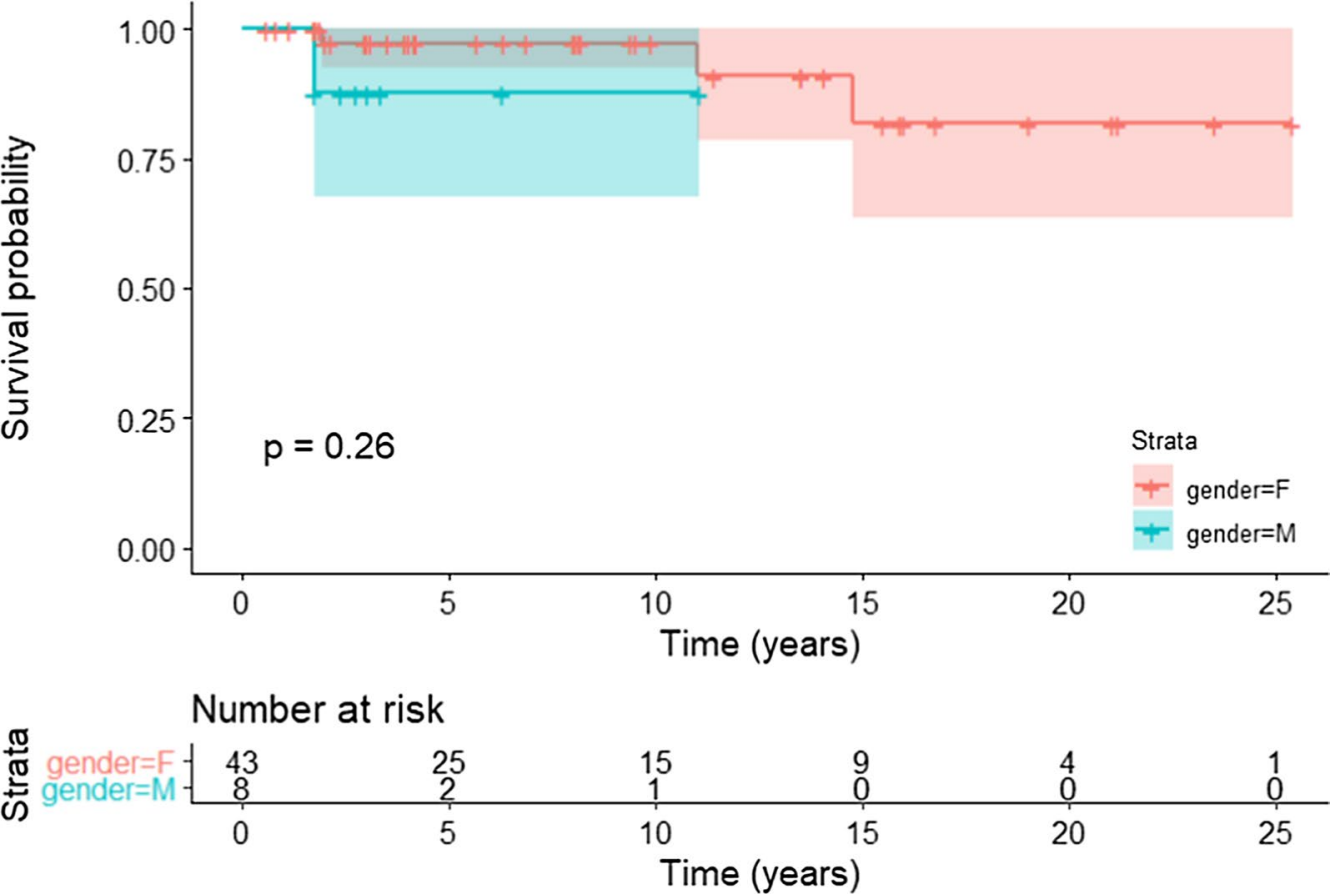
Kaplan Meier curve showing survival analysis post-transplant between male and female over time (years)

Patients were followed up for a median of 6.3 years (IQR = 2.9 -12.5), however, male patients were followed up for a median of 2.9 years (IQR = 2.2–4.1) vs a median of 8 years (IQR = 3–13.8) among females. At the end of follow-up, 16 (31.4%) were using Tacrolimus monotherapy while a combination of Tacrolimus and Mycophenolic acid was used in 11 (21.5%) patients. 24 (47%) patients were on combination therapy of Cyclosporin/ Mycophenolic or Cyclosporine alone. 1 (2%) of the patients developed insulin dependent Diabetes Mellitus, 2 (3.9%) developed CMV infection, 3 (5.8%) developed complications related the surgery such as pneumothorax/ pleural effusion. In addition, 3 (5.8%) developed T-cell mediated rejection and 2 (3.9%) had re-transplantation due hepatic artery thrombosis. There were 4 total deaths in our cohort, all greater than one year from transplant and were transplant-related (1 male, 3 females) with failed grafts. Log-rank test comparing survival among males and females was not significant (χ^2^(1) = 1.3, p = 0.26; Fig. 2). The overall probability of survival for our cohort is 100% (95% CI 100%,100%) at 1 year and 96% (95% CI 90%, 100%) at the 3, 5 & 10 year follow up.

## Discussion

Our study shows patients with PCLD undergo LT predominantly for symptom management, more specifically for the mass effect caused by the multiple hepatic cysts. Native MELD scores tend to be much lower than what would usually be needed to attract a deceased donor offer and therefore most attract a donor with the help of additional MELD exception points. Although most PCLD LT recipients were female, both genders had 5-year survival exceeding 90%.

PCLD is a disease characterized by presence of multiple cysts in the liver and is often associated with adult polycystic kidney disease (ADPKD). Isolated PCLD is usually associated with gene *SEC63* and *PRKCXH,* which code for special proteins involved in protein processing [5]. Once mutated, these proteins affect polycystin-1 and polycystin-2 which will lead the formation of cysts [6]. The main risk factors for having PCLD is having a family member with the disease, as it follows Mendelian mode of inheritance. Despite the similar genetic predisposition, the major risk factors for a massive hepatic cyst are female sex, exposure to female steroid hormone, pregnancy and exogenous hormone use such as oral contraceptives (OCPs) in postmenopausal women. This finding has led to the theory of probable hormonal regulation of these proteins leading to formation of hepatic cysts. This likely also explains why majority of PCLD patients are female, as estrogen stimulates cystolic receptors through a complicated signaling pathway leading to cyst proliferation [7].

There are multiple risk factors that play a pivotal role in predicting mortality in LT. In general, these factors are related to the pre-transplant kidney function, cardiac & pulmonary status [8]. Nonetheless, most LT programs have allocated exception points for PCLD patients taking this into account when it comes to prioritizing PCLD patients in LT waitlist [9]. Our study had a median age of 54 years which may have contributed to the excellent outcomes shown when compared to the European liver transplant registry group (ELTR) who had 1, 5, 10-year survival 89%, 85%, 77%, respectively, for patient with PCLD [10]. This finding may be related to having a relatively younger population that were otherwise healthy and potentially have contributed to the excellent outcomes seen for our population. However, there are modalities that can be tried before proceeding with LT.

Generally, patients with PCLD receive LT after exhausting medical therapy such as somatostatin analogues, radiological therapies such as sclerotherapy, cyst fenestration and lastly liver resection [11]. Despite the short acting half-life of naturally occurring somatostatin, synthetic long-acting somatostatins have been developed. Somatostatin interferes with biochemical signaling pathway inhibiting fluid secretion and have shown excellent results in patients with PCLD [12]. However, side effects may not be preferable to patients since it causes diarrhea and abdominal cramps. In the past, it has been suggested that combination of mammalian target of rapamycin (mTOR) inhibitors with somatostatin analogues may have better outcomes in comparison to somatostatin analogues monotherapy, but a recent trial suggested otherwise [13]. Aspiration with sclerotherapy is another treatment modality which considered safe and has regression and partial regression of 22% and 19% respectively [14]. With regards to cyst fenestration, patients’ needs to be selected carefully as it is preferred in patients with less severe disease and few, superficial, dominant hepatic cysts [15]. Lastly, patients may undergo hepatic resection with preservation of liver volume, however, this does not come without potential complications [16]. These complications can vary from anastomotic leak to massive ascites and should be considered in highly selected patients in which immunosuppressant is not desired. Therefore, it is reasonable to consider medical therapy with somatostatin analogues for patients with diffuse PCLD and to consider radiological and surgical treatments for patients with limited but large cysts before assessing for LT [17].

Liver transplantation has become a lifesaving procedure for many patients who meet criteria, however, this is limited by shortage of organ donors which makes organ allocation challenging for patients who need it the most [18]. PCLD patients often do not show the typical cirrhosis pathway sequelae from compensation to decompensation but rather have indications for LT due to symptom burden (cachexia, anorexia, abdominal distention) and to improve their quality of life. This leads to an ethical dilemma whether we subject otherwise healthy patients to lifelong immunosuppression only to improve their quality of life [19]. In addition to the above-mentioned challenges, this has led to increased wait times and waitlist mortality among other populations including patients with PCLD [21]. Recently, a similar situation was thought to be occurring with Hepatocellular carcinoma, and hence, the LT programs in Ontario decided to cap exception points at 30 and this may be an avenue to explore with PCLD given treatment modalities available and the excellent outcomes [22].

The strength of this study includes a large sample size from two established LT program that covers a population of 14 million people. However, there are important limitations of the current study that need to be highlighted. Firstly, the possibility of selection bias as PCLD patients are usually healthier compared to other patients with CLD may overestimate their survival. Also, our study population consists of mostly female patients limiting the generalizability to male patients, however, the nature of this disease dictates that it predominantly affects females. Secondly, no/missing data were available regarding surgical techniques, transfusion requirements, post-operative renal replacement therapy requirements, radiological and histopathological findings thus it was not included in our analysis. Lastly, our study included patients who had LT from 2000–2017 in two LT centers that have different surgical and therapeutic protocols which also may have affected survival however, surgical techniques and protocols have improved over time and so it is likely outcomes would at least be similar or even better.

## Conclusion

Patients with PCLD carry a high symptom burden requiring MELD-Na exception points to receive liver transplantation as many do not have high MELD-Na scores at the time of listing. Moreover, PCLD disproportionately affects women but overall long-term survival is excellent in both genders.

## Data Availability

Datasets used and/or analyzed during the current study are available from the corresponding author on reasonable request.

## Abbreviations

LT: Liver Transplant
PCLD: Polycystic Liver Disease
MELD-Na: Model of End Stage Liver Disease-Na
IQR: Interquartile Range
ADPKD: Adult Polycystic Kidney Disease
SLK: Simultaneous Liver-Kidney
CT: Computed Tomography
MRI: Magnetic Resonance Imaging
PCKD: Polycystic Kidney Disease
CMV: Cytomegalovirus
ELTR: European Liver Transplant Registry
CLD: Chronic Liver Disease
mTOR: Mammalian target of rapamycin
